# Exploration of the photothermal role of curcumin-loaded targeted carbon nanotubes as a potential therapy for melanoma cancer

**DOI:** 10.1038/s41598-024-57612-y

**Published:** 2024-05-02

**Authors:** Bahareh Kargar, Mehdi Fazeli, Zahra Sobhani, Saeid Hosseinzadeh, Aida Solhjoo, Amin Reza Akbarizadeh

**Affiliations:** 1 Department of Pathobiology, School of Veterinary Medicine, Shiraz University, Shiraz, Iran; 2 Department of Basic Sciences, School of Veterinary Medicine, Shiraz University, Shiraz, Iran; 3 Department of Drug and Food Control, School of Pharmacy, Shiraz University of Medical Sciences, Shiraz, Iran; 4 Department of Food Hygiene and Public Health, School of Veterinary Medicine, Shiraz University, Shiraz, Iran

**Keywords:** Multi-walled carbon nanotubes (MWCNTs), Curcumin, Drug delivery, Photothermal therapy, Cancer treatment, Nanobiotechnology, Melanoma, Drug delivery

## Abstract

In this research, the hydrophilic structure of multi-walled carbon nanotubes (MWCNTs) was modified by synthesizing polycitric acid (PCA) and attaching folic acid (FA) to create MWCNT–PCA–FA. This modified nanocomplex was utilized as a carrier for the lipophilic compound curcumin (Cur). Characterization techniques including TGA, TEM, and UV–visible spectrophotometry were used to analyze the nanocomplex. The mechanism of cancer cell death induced by MWCNT–PCA–FA was studied extensively using the MTT assay, colony formation analysis, cell cycle assessment via flow cytometry, and apoptosis studies. Furthermore, we assessed the antitumor efficacy of these targeted nanocomplexes following exposure to laser radiation. The results showed that the nanocomposites and free Cur had significant toxicity on melanoma cancer cells (B16F10 cells) while having minimal impact on normal cells (NHDF cells). This selectivity for cancerous cells demonstrates the potential of these compounds as therapeutic agents. Furthermore, MWCNT–PCA–FA/Cur showed superior cytotoxicity compared to free Cur alone. Colony formation studies confirmed these results. The researchers found that MWCNT–FA–PCA/Cur effectively induced programmed cell death. In photothermal analysis, MWCNT–PCA–FA/Cur combined with laser treatment achieved the highest mortality rate. These promising results suggest that this multifunctional therapeutic nanoplatform holds the potential for combination cancer therapies that utilize various established therapeutic methods.

## Introduction

Natural substances have gained attention as potential components of cancer treatment due to their anti-cancer properties and other favorable characteristics, such as low toxicity, minimal side effects, and structural similarities to synthetic medicines^1^. One such substance is Cur, obtained by extracting a hydrophobic polyphenol compound from the rhizome of Curcuma longa L. Cur has demonstrated promising anti-tumor properties against various cancers, including breast, colon, skin, pancreatic, and prostate cancer. Additionally, it exhibits anti-proliferative, antioxidant, anti-inflammatory, and apoptotic activities^2^. However, Curcumin’s low aqueous solubility, stemming from its hydrophobic nature, and its biological instability during metabolism in the human body limit its potential to reach therapeutic concentrations for effective anticancer outcomes^3^. To address these challenges, researchers have made significant strides in developing advanced formulations, primarily nanoformulations, as a primary strategy. A proposed mechanism by which Cur induces apoptosis in skin cancer involves the activation of caspases 3 and 8^3^. Furthermore, Cur has demonstrated its ability to inhibit melanoma cell migration and enhance apoptosis by down-regulating the JAK-2/STAT3 signaling pathways^4^.

Both in vitro and in vivo studies have shown that the use of nanocarrier systems to deliver Cur enhances its anti-proliferative properties and leads to regression in tumor growth compared to raw Cur^5^. Combining Cur-loaded nanoparticles with conventional chemotherapeutic drugs has shown improved treatment outcomes, with reduced side effects associated with the chemotherapeutic agents^6^. Nanoparticles (NPs) offer an attractive feature in enclosing therapeutic agents within functionalizable nanocarriers, forming diverse nanocomplexes for tumor therapy^7^.

Several nanotechnological therapeutic platforms, including carbon nanotubes (CNTs), liposomes, polymeric micelles, and emulsions, have emerged with significant potential for clinical applications, promising high efficacy and low toxicity in cancer treatment^8–10^. These nanocomplexes often rely on biocompatible matrix materials, such as polymers^11,12^ and silica^11,13^, or structural support and overall stability. However, despite their potential, challenges remain for the clinical use of these nano-therapeutics, including inherent nanotoxicity, time-consuming synthesis methods, and stability issues. There is a continued demand for innovative nanocomplexes that offer high biocompatibility and multifunctionality to achieve highly effective multi-modal cancer therapeutics.

Carbon nanotubes (CNTs) stand out as suitable delivery systems^14^, finding applications in various medical functions, particularly gene therapy, bioimaging, diagnostics, biosensors, and drug delivery systems^15^. Drug delivery systems increase the pharmacological and therapeutic profile and efficacy of drugs and reduce their distribution to non-target tissues^16^.

To render CNTs biocompatible, they often undergo modification processes with acid treatment being a common approach that introduces carboxyl groups on CNT walls, generating functionalized CNTs^17^. However, it's worth noting that this treatment has demonstrated cytotoxic properties in specific cell lines^18^. Alternatively, utilizing dendritic and hyper-branched biocompatible polymers like polycitric acid (PCA) to attach polymer chains to CNT walls enhances biocompatibility, interaction speed, solubility, and dispersion in biological fluids^19,20^. PCA, being a hyper-branched polymer, offers an extremely biocompatible surface^21^. PCA has been designed to create biological scaffolds and grow CNT hydrophilicity, decreasing their accumulation and polydispersity size, and thereby reducing their toxicity^22^. Importantly, the PCA polymerization process does not yield pollutant elements^23^. The nanoplatform MWCNT–PCA results in the effective prevention of Cur from degradation and fast release of Cur in a disordered state.

To enhance therapeutic efficacy and minimize adverse side effects, it is essential to achieve specific targeting of cancer sites. The inclusion of folic acid (FA) on the surface of MWCNT–PCA can serve as a targeted delivery mechanism for these nanocomplexes, facilitating their delivery to tumor locations^24–26^. These nanocomplexes, comprising MWCNTs, PCA, FA, and Cur, hold promise as a potential drug delivery platform for clinical applications. Photothermal therapy (PTT) presents a successful approach for eliminating solid tumors, as local hyperthermia induced by photothermal conversion under NIR laser irradiation irreparably damages tumor cells^27–29^. NIR-laser-mediated PTT offers advantages such as minimal invasiveness, better preservation of normal tissue, deeper penetration, quicker recovery, and fewer complications compared to earlier treatments^30,31^. Researchers have developed various well-designed agents, including carbon-based nanocomposites, metal-based nanomaterials^32^, and organic NPs^33^, for photothermal treatment.

In this study, our objective is to prepare and evaluate targeted carbon nanotubes containing Cur- (MWCNT–PCA–FA/Cur NPs) and assess their in vitro cytotoxicity effects and fate. We utilize NIR laser irradiation to magnify the impact of MWCNT–PCA–FA/Cur on inhibiting tumor cell growth. Our research’s primary contribution involves the exploration of MWCNT–PCA–FA/Cur in various cellular processes, including cell cycle, apoptosis, and colony formation. We anticipate that our findings will contribute to the advancement of MWCNT–PCA–FA/Cur NPs as effective drug delivery systems, harnessing the potential of CNTs as efficient nanocarriers for drug delivery.

In the literature, a few studies evaluate the combination effect of Cur and NIR irradiation on cancerous cells^34–36^. In those studies the drug carrier is different from MWCNT–PCA–FA and the cytotoxicity findings are limited to MTT assay. In the current study, various cellular techniques are employed to understand the fate of insoluble molecules of Cur with and without carrier on cellular growth and death.

## Materials and methods

Multi-walled carbon nanotubes (MWCNTs) with a purity exceeding 95%, outer diameter (OD) ranging from 20 to 30 nm, and lengths between 10 and 30 µm, were procured from the Iranian Nanomaterial Pioneers company. Citric acid was obtained from Chem-Lab NV, Belgium. Chemicals including cyclohexane, tetrahydrofuran (THF), sulfuric acid (95–97%), nitric acid (65%), potassium dihydrogen phosphate, sodium chloride, Tween-80, Curcumin (Cur), N-(3-(dimethylamino)propyl)-N′-ethyl carbodiimide hydrochloride (EDC), and N-hydroxysuccinimide (NHS) were sourced from Merck, Germany. Acetone and methanol were acquired from Caledon-Canada. Folic acid (FA) was kindly provided by Exir Pharmaceutical Factory, Iran. All chemicals used in this study were of analytical grade.

### Oxidation of MWCNTs

MWCNTs were oxidized following established methods from the literature. Briefly, 1 g of MWCNTs was mixed with 20 ml of concentrated HNO3 and H2SO4 (in a 1:3 ratio). The resulting dispersion underwent sonication for 30 min and was refluxed for 24 h at 120 °C. The black suspension was subsequently diluted with 1 L of distilled water, filtered, and washed with deionized water until reaching a pH of 6. The oxidized MWCNTs (OCNT) were then dried in an oven^37,38^.

### Functionalization of OCNT with PCA

Polymerization of polycitric acid (PCA) on the surface of OCNT was carried out following a previously established protocol^38^. Specifically, 0.05 g of OCNTs and 5 g of monohydrate citric acid were combined in a polymerization ampoule equipped with a magnetic stirrer and a vacuum inlet. The mixture was heated to 120 °C while stirring for 30 min. The reaction temperature was gradually raised to 140 °C and 160 °C for 1 h and 1.5 h, respectively, under vacuum conditions. Subsequently, the product was allowed to cool to room temperature, dispersed in THF, and any residual citric acid was precipitated in cyclohexane. The resultant product underwent multiple washes with THF and was dried in an oven at 50 °C for 24 h. The resulting material was termed MWCNT–PCAs^21^.

### Conjugation of folic acid to MWCNT–PCA

To facilitate the conjugation of folic acid (FA) to the ends of PCA chains on the CNT surface, 0.06 g of MWCNT–PCA was introduced into 5 ml of phosphate buffer with a pH of 6. Then, 0.07 mmol of EDC and 0.115 mmol of NHS were added to the mixture. The reaction was stirred for 30 min at room temperature in the dark. Separately, 0.005 mmol of FA was dissolved in 4 ml of phosphate buffer with a pH of 8. The activated FA was added dropwise to the MWCNT–PCA mixture. The reaction was left to proceed for 2 h under dark conditions. The resulting MWCNT–PCA–FA nanoparticles were dialyzed for 24 h and subsequently freeze-dried^39^.

### Curcumin loading on MWCNTs –PCA-FA

To incorporate Cur into the functionalized nanocarrier surface, various weight ratios of the nanocarrier to the drug were mixed, including ratios of 1:0.1, 1:0.2, 1:0.5, 1:1, 1:2, 1:3, 1:4, and 1:5 (w/w). Specifically, different quantities of Cur solution (0.2 mg/ml) in acetone were added dropwise to 0.2 mg/ml of MWCNT–PCA–FA in distilled water, thus generating specific weight ratios of Cur to the carrier. The mixtures were shaken overnight at 25 °C^40^. Unloaded drugs were separated through centrifugation at 25,200 g for 15 min, followed by rinsing the MWCNT–PCA–FA/Cur precipitate with methanol to remove excess Cur. The quantity of unloaded drug in the supernatant was determined by UV–Vis spectroscopy at 422 nm. The MWCNT–PCA–FA/Cur nanocomposites were then vacuum-dried for 12 h. The loading amount (LA%) and loading efficiency (LE%) were calculated using the following equations^41,42^.

1 $${\text{LA}}\% = \left( {\text{amount of loaded drug / total weight of nanocomposites}} \right)\; \times \;{1}00$$

2 $${\text{LE}}\% = \left( {\text{amount of loaded drug / initial amount of drug}} \right)\; \times \;{1}00$$

### Physical and chemical characterization of nanoparticles

MWCNT–PCA, MWCNT–PCA–FA, and MWCNT–PCA–FA containing Cur were characterized by different analysis techniques such as Transmission Electron Microscopy (TEM), Thermogravimetric analysis (TGA), and UV–Vis spectroscopy^39^.

### Curcumin release study

The investigation of drug release was conducted at three distinct pH levels: pH 7.4 and pH 6.8 within a phosphate-buffered saline (PBS) environment, and pH 5.0 within an acetate buffer. For this purpose, 0.2 mg of MWCNT–PCA–FA/Cur was dispersed in 2 ml of PBS (pH 7.4 and pH 6.8) and acetate buffer (pH 5.0) solutions containing 0.5% Tween 80. Subsequently, the vessels were placed within a shaker incubator operating at 37 °C. At specific time intervals of 0.5, 1, 2, 4, 6, 8, 24, 48, 72, 96, and 120 h, the samples underwent centrifugation at 16,100 g for 10 min at 4 °C. The clear supernatants were meticulously collected, and precisely 2 ml of fresh medium was introduced in their place. The quantification of released drug was achieved through absorbance measurements at 422 nm. This procedure was performed in triplicate, and the cumulative percentage of Cur released was subsequently determined^43^.

### Hemolysis assay

In the initial step, human red blood cells (RBCs) were obtained through centrifugation at 800 g for 10 min at 4 °C, followed by the removal of blood plasma and the surface layer. Subsequently, the RBCs were subjected to five wash cycles using phosphate-buffered saline (PBS) (specifically Dulbecco's PBS, Gibco) at pH 7.4. The erythrocytes were then diluted tenfold, and the resulting suspension was transferred to sterile 2 ml microtubes. A two-fold serial dilution of Cur, as well as the nanocomposite with and without Cur, was initiated, starting at a concentration of 50 μg/ml in a PBS solution. These samples were incubated at 37 °C for 2 h within a shaker incubator. Subsequently, the microtubes were centrifuged at 200 g for 10 min. The hemolysis percentage was determined by measuring the absorbance of the supernatant at 540 nm. Positive and negative control samples were prepared by adding 1 ml of distilled water and PBS to 0.2 ml of RBC solution, representing 100% and 0% hemolysis, respectively. The hemolysis percentage of RBCs was calculated using the following equation^44^. All steps in the hemolysis assay were repeated three times to ensure the accuracy and reliability of the results.

3 $${\text{Hemolysis\% }} = { }\frac{{{\text{absorbance of sample }}{-}{\text{ absorbance of negative control}}/}}{{{\text{absorbance of positive control }}{-}{\text{ absorbance of negative control}}}}\; \times \;100$$

### Cell lines and cell culture

Skin melanoma cancer cell lines (B16F10) and Normal Human Dermal Fibroblast cell lines (NHDF) were procured from the Stem Cell Technology Institute of Tehran, Iran. B16F10 cells were cultured in RPMI 1640 medium (Bio Idea, Iran), while the NHDF cell line was cultivated in DMEM with high glucose (Bio Idea, Iran). Both culture media were supplemented with 10% fetal bovine serum (FBS; Gibco, USA) and 1% penicillin–streptomycin (Logan, UT, USA). The cell cultures were maintained at a constant temperature of 37 °C in a humidified CO2 incubator^45^.

### Cytotoxicity study

The cytotoxic activity of all synthesized compounds, including MWCNT–PCA–FA, free Cur, and Cur-loaded nanocomposites, was assessed using the standard 3-(4,5-dimethylthiazol-yl)-2,5-diphenyl-tetrazolium bromide (MTT) assay following a well-established protocol^46^. Cells were dissociated from culture using a 0.5% trypsin/EDTA solution (Gibco, USA) and seeded at a density of 5000 cells per well within 96-well microplates. These cells were subsequently incubated at 37 °C for 24 h prior to viability evaluation. Each cell line was exposed to six distinct concentrations spanning from 0.625 to 20 µg/ml, with each concentration tested in triplicate^44^. Three wells without any treatment were employed as negative^46^. Three wells without any treatment were employed as negative controls. After 24 h, the culture media were aspirated and replaced with 20 µL of an MTT solution (5 mg/ml in PBS). They were allowed to incubate for 3 h at 37 °C within an incubator, facilitating the formation of insoluble formazan crystals. To dissolve these formazan crystals, 100 µL of DMSO was added. The absorbance of the wells was quantified using a microplate ELISA reader (BD, USA) at 570 nm. A concentration-versus-viability plot was constructed, and the IC50 value, representing the concentration causing 50% growth inhibition, was determined^47^.

### Colony analysis

A clonogenic assay, also referred to as a colony formation assay, is an in vitro cell survival assessment that hinges on the ability of a single cell to propagate into a colony, typically defined as having a minimum of 50 cells. This assay effectively scrutinizes each cell within the population for its potential to undergo unlimited divisions. It is the preferred technique for quantifying reproductive cell mortality subsequent to treatment with cytotoxic substances^48^. Cells were cultivated in 6-well plates at a density of 1000 cells per well. After a 48-h incubation period, varying concentrations of nanoparticles (6.25 and 12.5 μg/ml) of MWCNT–PCA–FA and MWCNT–PCA–FA/Cur, along with free Cur, were introduced to both the B16F10 and NHDF cell lines. Following a 24-h treatment interval, the medium containing the nanoparticles was replaced with a fresh medium.

Over ten days, the culture medium was refreshed every three days until colonies became visible. Subsequently, the well’s media were aspirated, and the cells were washed with PBS and then stained with 0.5% crystal violet. Stained colonies within each well were enumerated, and those containing more than 50 cells were selected for the calculation of plate efficiency (PE) and the colony surviving fraction (SF) using the following equations^48^.

4 $${\text{PE}}:\frac{{\text{Number of colonies formed }}}{{\text{Number of cells implanted in the well}}}\; \times \;100$$

5 $${\text{SF}}:\frac{{\left( {{\text{PE}}} \right){\text{ treated cells}}}}{{\left( {{\text{PE}}} \right){\text{ control cells}}}}\; \times \;100$$

### Cell cycle analysis

To perform the cell cycle assay, a 6-well plate was utilized to seed 5 × 10^5^ B16F10 cells in 2 ml of complete culture medium, followed by a 24-h incubation period. Subsequently, the cells were treated with three compounds (MWCNT–PCA–FA, free Cur, and Cur-loaded nanocomposites) and were incubated for the appropriate duration. After incubation, the cells were trypsinized, collected, and fixed in 0.5 ml of 70% EtOH. A centrifugation step at 500 g for 2 min was employed to pellet the cells, and the resulting cell pellet was then resuspended in 0.5 ml of PBS containing 0.25% Triton X-100, followed by a 15-min incubation on ice. Following this incubation, a repeat centrifugation step was performed, and the supernatant was discarded. The cell pellet was resuspended in 0.5 ml of PBS containing 10 μg/ml Ribonuclease A (Sigma-Aldrich, Germany) to ensure selective DNA staining. Subsequently, DNA was stained using 20 μg/ml PI (Sigma-Aldrich, Germany). The stained cells were transferred to Fluorescence-Activated Cell Sorting (FACS) tubes and incubated at room temperature in the dark for 30 min. Finally, the cells were prepared for analysis using the FACS flow cytometer from BD Biosciences, with data analysis performed using FlowJo software^49^.

### Apoptosis analysis

To assess apoptosis induced by MWCNT–PCA–FA, free Cur, and Cur-loaded nanocomposites on the B16F10 cell line, an Annexin V/PI apoptosis detection kit (Biolegend) was employed. As per reported protocols^48,49^, cells were seeded at a density of 5 × 105 cells per 2 ml of complete culture medium in a 6-well plate and incubated overnight at 37 °C in a CO2 incubator. Subsequently, the supernatant was aspirated, and cells were treated with the desired compound concentrations. An untreated sample served as a negative control. After 24 h of incubation, adherent cells were trypsinized, collected, and washed with cold 1 × PBS. Pellets were dissolved in 100 μl of binding buffer and transferred to polystyrene tubes (BD Biosciences, USA). They were then stained with 5 μl of Annexin V-FITC and 5 μl of PI, followed by a 15-min incubation at room temperature in the dark. Subsequently, 5 μl of propidium iodide (PI) and 400 μl of 1 × binding buffer were added to each tube. The samples were analyzed by flow cytometry (BD Biosciences) immediately after adding PI. Data were analyzed using FlowJo software^50^.

### NIR laser-induced temperature elevation

The NIR laser-induced temperature elevation of MWCNT–PCA–FA/Cur was characterized by subjecting 2 ml aqueous dispersions of NPs to laser irradiation (output power = 1 W, wavelength = 808 nm) across a range of concentrations for 10 min. During laser irradiation, the solution temperature was dynamically recorded at 1-min intervals using a digital thermometer (MasPower, Germany)^51,52^.

### In vitro cytotoxicity and photothermal efficiency

To characterize the cytotoxicity of the synthesized NPs following laser irradiation, the B16F10 cell line was exposed to varying concentrations of CNT-PCA-FA, free Cur, and Cur-loaded nanocomposites (0.625–20 µg/ml). To assess the photothermal efficiency of the nanoparticles, cells were irradiated with a NIR 808-nm laser at a power of 1 W for 10 min, followed by an additional 24-h incubation. For the MTT assay, 20 µl of an MTT solution (5 mg/ml in PBS) was introduced into each well to measure cell survival. Incubation continued for 3 h at 37 °C to develop insoluble formazan. The formazan crystals were then dissolved using 100 µl of DMSO, and the absorbance of the soluble formazan product was measured using a multimode reader (Cytation 3, Biotek) at 570 nm. The percentage of cell viability was calculated^53,54^.

### Statistical analysis

All experiments were conducted in triplicate, and the results were presented as the mean ± standard deviation for each analysis. Statistical analysis of the experimental data was performed using one-way ANOVA with GraphPad Prism 9.0 software (GraphPad Software Inc.).

## Result and discussion

### Preparation and characterization of MWCNT–PCA–FA/Cur

**Figure 1:**
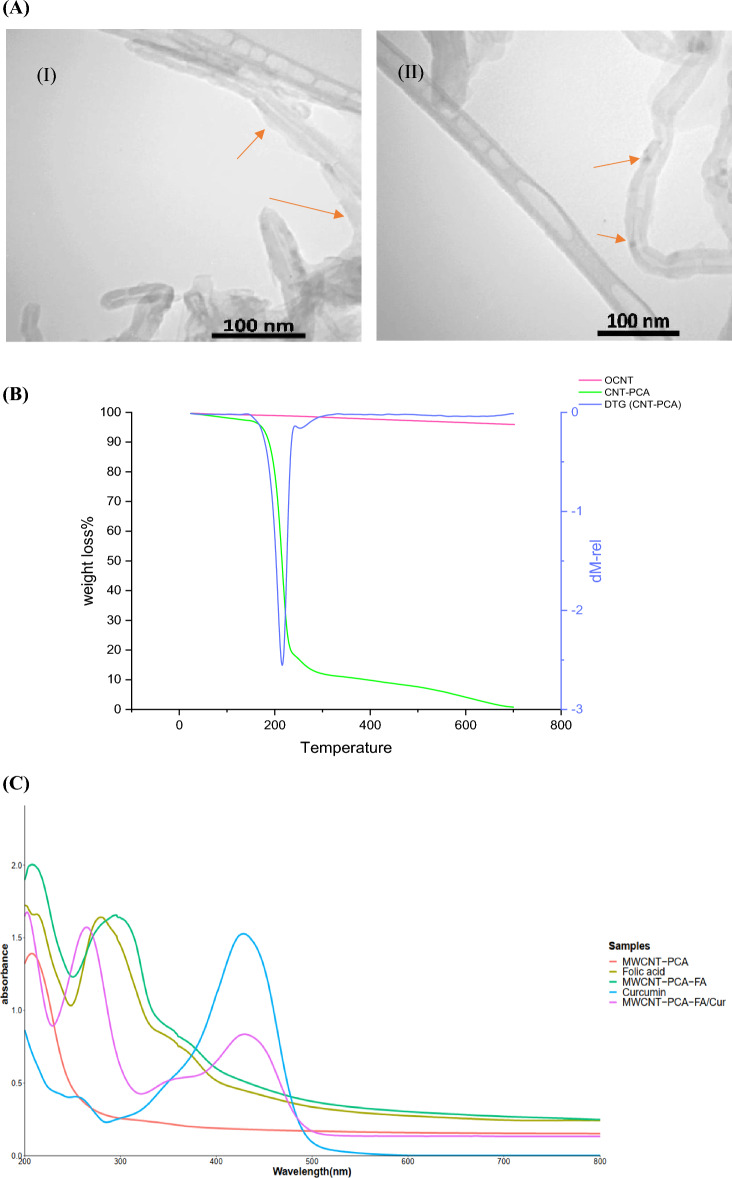
(**A**) The TEM image of the (**A**) (I) OCNT-PCA nanoparticles. The arrows show a coating of poly citric acid on the surface of the OCNT, and (**A**) (II) Cur are black spots on the OCNT-PCA-FA. (**B**) TGA thermograms for OCNT and MWCNT–PCA, DTG thermograms for MWCNT–PCA. (**C**) UV spectrum of FA, MWCNT–PCA, MWCNT–PCA–FA, free Cur, MWCNT–PCA–FA/Cur.

The morphologies of OCNT, MWCNT–PCA–FA, and MWCNT–PCA–FA/Cur particles were visualized via transmission electron microscopy (TEM) as depicted in Fig. 1A. Figure 1A (I) highlights the polymer layer enveloping the OCNT. Additionally, the presence of Cur on the MWCNT–PCA–FA surface is evident in Fig. 1A (II). The thermal stability of OCNTs and MWCNT–PCA was assessed through thermogravimetric analysis (TGA) and derivative thermogravimetric analysis (DTG), as shown in Fig. 1B. OCNT nanoparticles exhibited stability up to temperatures of 700 °C. TGA analysis revealed that approximately 77% of the weight loss was attributed to the PCA polymer. This substantial presence of hydrophilic PCA polymer on the CNT surface contributes to the stable dispersion of OCNT particles in aqueous media. The DTG graph (Fig. 1B) also demonstrates a noticeable weight reduction of nanoparticles at a temperature of 218 °C.

Figure 1C displays the UV–Vis spectra for OCNT, MWCNT–PCA–FA, MWCNT–PCA–FA/Cur, and free Cur within the wavelength range of 200–800 nm. The maximum absorption of FA occurs at 280 nm. Neither FA nor Cur absorptions on the NPs interact with any components, allowing their respective maximum wavelengths at 280 nm and 422 nm to be used for the quantification of FA and Cur in MWCNT–PCA–FA/Cur.

### Curcumin loading on MWCNTs-PCA analysis

**Figure 2:**
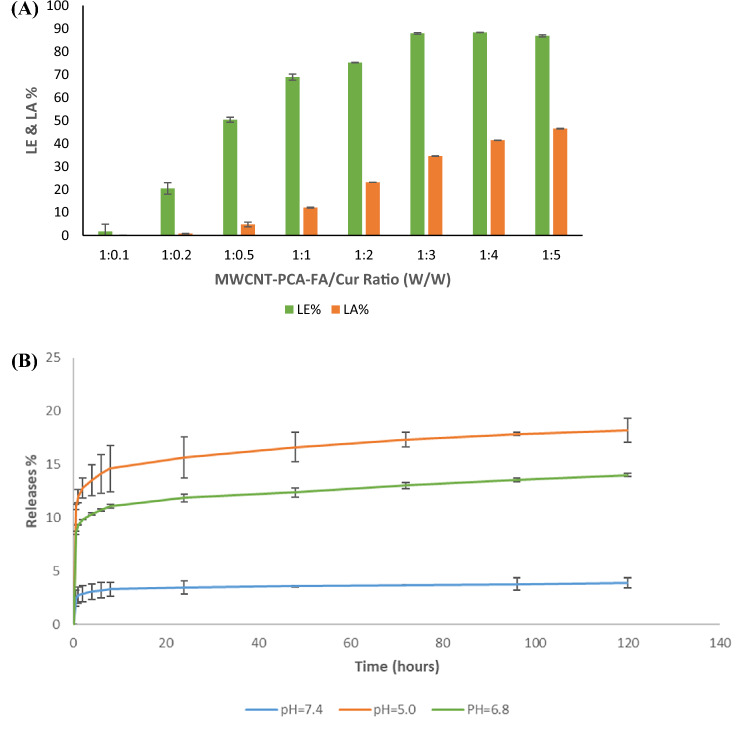
(**A**) The LE% and the LA% of Cur on MWCNT–PCA–FA (n = 3). (**B**) The release process of Cur from MWMCNT–PCA–FA/Cur at three different pHs (n = 3).

The primary objective of this section is to assess the efficiency of Cur loading onto MWCNT–PCA–FA nanoparticles. In pursuit of this goal, the Loading Amount (LA%) and Loading Efficiency (LE%) were analyzed for eight different MWCNT–PCA–FA to Cur ratios, specifically 1:0.1, 1:0.2, 1:0.5, 1:1, 1:2, 1:3, 1:4, and 1:5 (w:w). As depicted in Fig. 2A, an increase in the drug quantity resulted in higher LA% and LE%. The LE% remained relatively consistent at the ratios of 1:3, 1:4, and 1:5. The highest observed LE% was 88.3% ± 1.17, achieved at a 1:4 ratio of MWCNT–PCA–FA to Cur. The primary mechanisms contributing to Cur LE% included π-π stacking between the surface of MWCNTs and Cur, along with the formation of hydrogen bonds between Cur and the hyper-branched PCA on the surface of MWCNTs.

In comparison with other nanoparticles, such as mesoporous silica^55^, graphene^56^, liposomes^57^, and polymeric micelles^12^, MWCNT–PCA–FA nanoparticles demonstrated a notably high capacity for Cur loading. However, it's worth noting that while the LE% was high at the 1:3, 1:4, and 1:5 ratios, these nanoparticles exhibited limited stability in aqueous environments and underwent rapid precipitation. In the 1:2 ratio, MWCNT–PCA–FA/Cur remained stable for an extended period, up to 1 month. Consequently, this ratio was selected as the optimal condition for Cur loading, with corresponding LA% and LE% values of 23.12% ± 1.22 and 75.20% ± 1.17, respectively.

### In vitro release of curcumin

Due to its favorable characteristics, including nano-size, appropriate chemical structure, and pH sensitivity, MWCNT–PCA–FA serves as an ideal carrier for drug delivery systems. Cur-loaded functionalized nanotubes can be absorbed by many human cancer cells through endocytosis. Following absorption, Cur is released from MWCNTs into the cells due to the acidic conditions within endosomes^58^. The release of Cur from MWCNT–PCA–FA/Cur was studied at 37 °C under three distinct pH conditions, specifically 5.0, 6.8, and 7.4, for a duration of up to 120 h (Fig. 2B). The drug release exhibited a pH-dependent pattern, with notably faster release rates observed in acidic environments. This phenomenon can be attributed to the amino group in Cur being more prone to protonation in acidic conditions, thereby enhancing its solubility^59^. Given that tumor cells often reside within a more acidic microenvironment (pH = 4.5–6.5) compared to normal tissues, pH-triggered drug release is preferred to minimize undesired toxicity to normal tissues during in vivo drug circulation^60^. The release amount at acidic pH exceeded that at neutral pH, a critical factor for many cancer drugs^41^. As observed in Fig. 2B, the cumulative release of Cur rapidly reached 15.63% within the initial 24 h, stabilizing at this level at pH 5.0. In contrast, only approximately 3.4% of Cur was released within the first 12 h at pH 7.4, with cumulative release not exceeding 3.9% over 120 h.

In Fig. 2, the release values at three pH values, namely 5.0, 6.8, and 7.4, are compared through One-Way ANOVA analysis, and a significant difference was determined by the Tukey test that yields *p* < 0.0001.

Throughout this study, statistical analysis of data is done by GraphPad Prism 8 software. Data are expressed as mean ± SD. All the experiments are repeated at least three times, and the data are analyzed using a one-way ANOVA test. *p* < 0.05 is considered significant.

### Results of hemolysis assay

**Figure 3:**
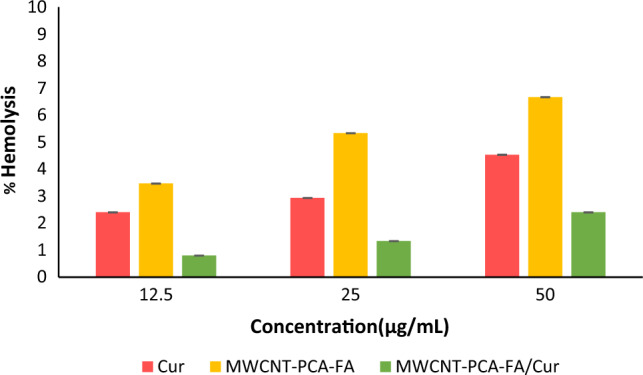
Effect of Cur and Cur-loaded nanocomposite on RBC membrane integrity. Data are shown as mean ± S.D of three independent assays. MWCNT–PCA–FA/Cur: *p*-value between three groups: *p < 0.05.

To evaluate the hemocompatibility of the synthesized nanoparticles, a hemolysis assay was conducted, a fundamental assessment in the realm of biomedical and pharmaceutical research^44^. Significant hemolytic activity was observed when examining three concentrations (12.5, 25, and 50) of nanocomposites and free Cur. As depicted in Fig. 3, higher levels of hemolysis were induced by both Cur and MWCNT–PCA–FA individually across all three concentrations, in comparison to MWCNT–PCA–FA/Cur. This observation indicates a reduction in the toxicity of Cur and MWCNT–PCA–FA when the drug is loaded onto the carrier. Notably, the lowest hemolytic activity was detected in MWCNT–PCA–FA/Cur (0.8% hemolysis at 12.5 μg/ml), whereas free Cur and MWCNT–PCA–FA nanocomposites exhibited higher hemolysis rates at 2.4% and 3.46%, respectively. Remarkably, the supernatant surrounding MWCNT–PCA–FA/Cur remained clear across all concentrations, affirming the excellent hemocompatibility of MWCNT–PCA–FA/Cur, which positions it as a promising candidate for targeted intravenous drug delivery systems.

### In vitro cytotoxicity of nanocomposite on B16F10 and NHDF cells

**Figure 4:**
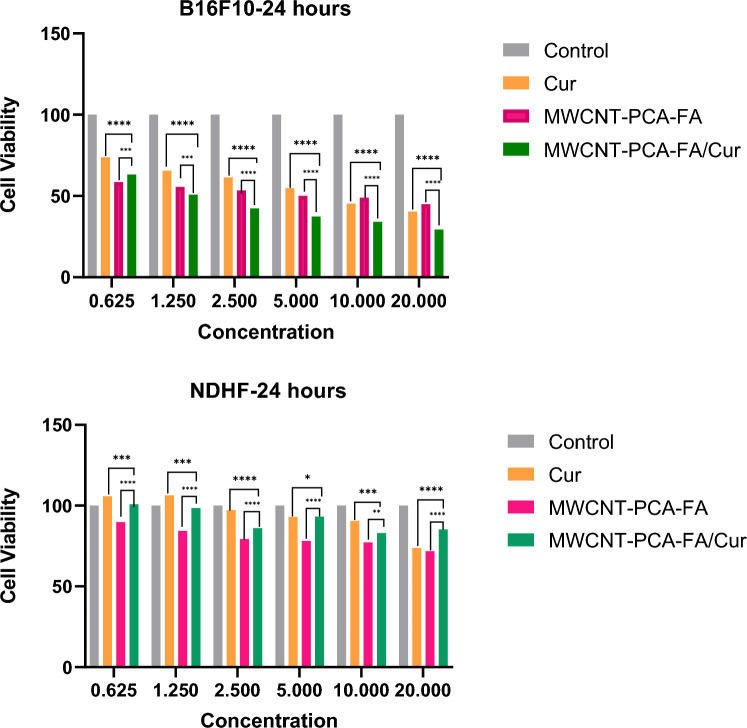
Cell viability study of Cur and the nanocomposites on B16F10 and NHDF cell lines by respective concentrations at 24 h. The results of three separate experiments are presented as mean ± S.D. MWCNT–PCA–FA/Cur: *p < 0.05, **p < 0.01, ***p < 0.001, and ****p < 0.0001.

The potential toxicity of nanoparticles is a crucial consideration in their biomedical and pharmaceutical applications. Ensuring that designed compounds do not adversely affect biological systems is of utmost importance. Therefore, we assessed the inhibition of tumor growth by free Cur, MWCNT–PCA–FA, and MWCNT–PCA–FA/Cur on B16F10 and NHDF cell lines. Over a 24-h treatment period, we employed the MTT test to detect any metabolic changes induced by the produced nanocomposites, indicative of apoptosis or proliferation. Furthermore, we compared the effectiveness of free Cur and MWCNT–PCA–FA in treating melanoma cancer with that of MWCNT–PCA–FA/Cur in our study. As illustrated in Fig. 4, all compounds inhibited the proliferation of B16F10 cells compared to the control group. All combinations exhibited favorable cytotoxic effects on B16F10 cells in a dose-dependent manner (ranging from 0.625 to 20 µg/ml). Both nanocomposites and free Cur had a toxic impact on cancerous melanoma cells (B16F10). Remarkably, they had no significant effect on normal cells (NHDF), attributed to the strong selectivity of the designed compounds for cancerous cells over non-cancerous ones. Among all compounds, MWCNT–PCA–FA/Cur demonstrated greater cytotoxicity than free Cur and CNT-PCA-FA at equivalent doses. This heightened cytotoxicity can be attributed to the enhanced cellular penetration of MWCNT–PCA–FA/Cur, facilitated by the functional groups and FA on the MWCNT–PCA–FA surface. The IC50 values for free Cur and MWCNT–PCA–FA/Cur were 6.3 and 1.26 μg/ml, respectively, underscoring the superior inhibitory effects of free Cur and MWCNT–PCA–FA/Cur on B16F10 cells.

As depicted in Fig. 4, MWCNT–PCA–FA exhibited toxicity toward both tumorigenic and non-tumorigenic cell lines. Several factors have been stated for the toxicity of carbon nanotubes, the most important is their hydrophobic structure and bundle formation in the cell wall, which cause cell damage, but by surface modification through hydrophilic polymer such as polycitric acid, this problem was solved. The toxicity of drug-free nanocomposite (MWCNT–PCA–FA) on normal cells (NHDF) is much less compared to the cancer cells (B16F10), which can indicate less penetration of this carrier into healthy cells. Since the FA receptors are more expressed in the cancer cells, the penetration of the NPs containing FA molecules to these cells is more than in healthy cells. After penetration, ROS generation and lysosome damage are the main mechanisms of cell death induced by carbon nanotubes^61^. Loading Cur onto these nanoparticles causes a reduction in the toxicity of NPs on normal cells (NHDF). These findings emphasize the safe and biocompatible nature of the MWCNT–PCA–FA nanocomposite as a drug delivery system, thanks to the functional groups on the MWCNT surface. In summary, our results demonstrated that MWCNT–PCA–FA/Cur reduced cell growth compared to free Cur and MWCNT–PCA–FA, highlighting its potential in cancer therapy.

### Colony study

**Figure 5:**
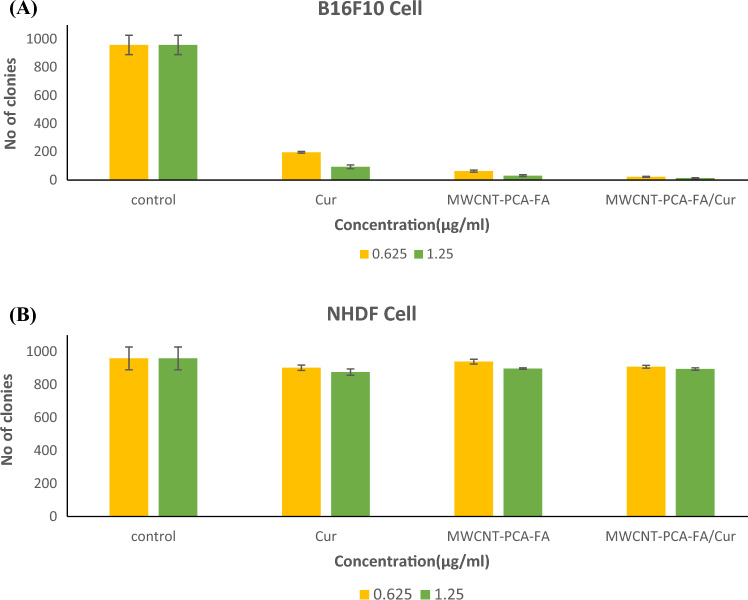
The graph shows the cells exposed to free Cur and carbon nanocomposites with and without Cur and colony number of (**A**) B16F10 and (**B**) NHDF cell lines after exposure to different concentrations of NPs. Three independent experiments were done in the three repetitions for each concentration. Untreated cells were used as the control for comparison. Data are shown as mean ± S.D of three independent assays. MWCNT–PCA–FA/Cur: *p*-value between three groups: *p < 0.05.

Another confirmatory test for assessing the cytotoxicity of the designed compounds involved colony analysis. The capacity of cancer cells to reproduce, as measured by their clonogenicity or the ability of a single-cell suspension to generate progeny, was evaluated. Colony formation inhibition by designed compounds was assessed on B16F10 and NHDF cell lines. The results for each cell line were compared to the negative control group in a dose-dependent manner. As depicted in Fig. 5A,B, the most pronounced cytotoxic effect was observed in B16F10 cells when compared to normal cells, which was associated with the acidic pH in the vicinity of the cancerous cell line. In fact, among all compounds, the least colony formation was observed with the MWCNT–FA–PCA/Cur nanocomposite (at a concentration of 1.25 µg/ml). This nanocomposite induced alterations in cell monolayers, resulting in patches devoid of cells and morphological abnormalities. However, colonies were observed in the case of normal cells (NHDF), indicating their resistance to these compounds.

### Cell cycle analysis via flowcytometry

**Figure 6:**
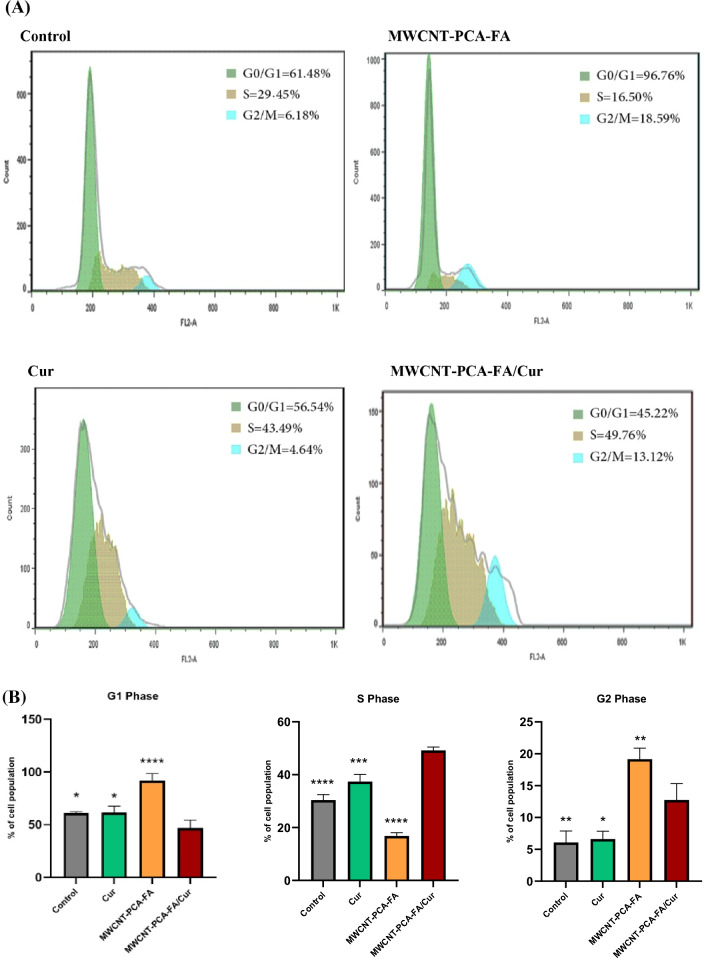
Treatment with MWCNT–PCA–FA, Cur, and MWCNT–PCA–FA/Cur modifies B16F10's cell cycle pattern. Cells in (**A**) were treated for 24 h with free Cur and nanocomposites before being fixed with 70% ice-cold ethanol. PI staining was used to analyze flow cytometry data to detect DNA content. (**B**) Quantification of the G0/G1, S, and G2/M phases in the B16F10 cells. The results of three separate experiments are presented as mean ± S.D. MWCNT–PCA–FA/Cur: *p 0.05, **p < 0.01, ***p < 0.001, and ****p < 0.0001.

Uncontrolled and dysregulated cell proliferation stands as a hallmark feature of cancer. This section delves into the impacts of MWCNT–PCA–FA, free Cur, and Cur-loaded nanocomposites on the cell cycle, establishing a crucial link between growth inhibition and cell cycle arrest. Cell cycle arrest analysis was meticulously conducted utilizing the flow cytometry method to dissect the distribution of DNA duplication within the cell cycle—specifically at the G1, S, and G2/M phases. We meticulously evaluated the effects of various treatments on B16F10 cells arrested at distinct cell cycle phases. To this end, B16F10 cells underwent a 24-h treatment regimen with three different compounds, compared to untreated cells. As vividly portrayed in Fig. 6, in stark contrast to the control group, MWCNT–FA–PCA treatment led to a remarkable accumulation of cells within the G1 phase, witnessing an escalation from 61.48% to a staggering 96.76%. Simultaneously, the percentage of cells within the S phase underwent a substantial decline, plummeting from 29.45% to a mere 16.50%. In striking contrast, Cur treatment ushered in a pronounced increase in the proportion of B16F10 cells occupying the S phase, witnessing a surge from 29.45% to an impressive 43.49%. This marked increase in cell population within the S phase coexisted with notable reductions in both G0/G1 and G2/M phases, compelling evidence of Cur-induced cell cycle arrest. Furthermore, as the 24-h incubation period concluded with the different compounds, MWCNT–FA–PCA/Cur exerted a significant influence. It incited a substantial surge in the cell population within the S phase, escalating from 29.45% to a formidable 49.76%, in comparison to the control group. This robust observation definitively denotes that MWCNT–FA–PCA/Cur induced potent cell cycle arrest. This compelling outcome is typified by the upsurge in cell population within the S phase and the corresponding reduction in G0/G1 phases. Disruption of the cell cycle holds profound implications for tumorigenesis. Accordingly, targeting the cell cycle pathway emerges as a robust strategy within the realm of cancer therapy. Cell cycle arrest can offer tumor cells an opportunity for DNA damage repair or, conversely, instigate the apoptotic cascade, culminating in programmed cell death^62^. Our findings resolutely affirm that MWCNT–FA–PCA/Cur orchestrated a pronounced shift of B16F10 cells from the G1 phase to the S phase of the cell cycle. The marked accumulation of cells at the S phase alludes to the occurrence of apoptotic cell death preceding DNA replication during the G2/M phase. MWCNT–FA–PCA/Cur adeptly initiated programmed cell death, substantiated by the conspicuous elevation in the proportion of cells within the S phase. In light of these findings, our study propounds MWCNT–FA–PCA/Cur as a formidable contender for the treatment of melanoma cells, underpinned by its capacity to trigger robust cell cycle arrest.

### Induction of apoptosis studied by Annexin V-FITC assay

**Figure 7:**
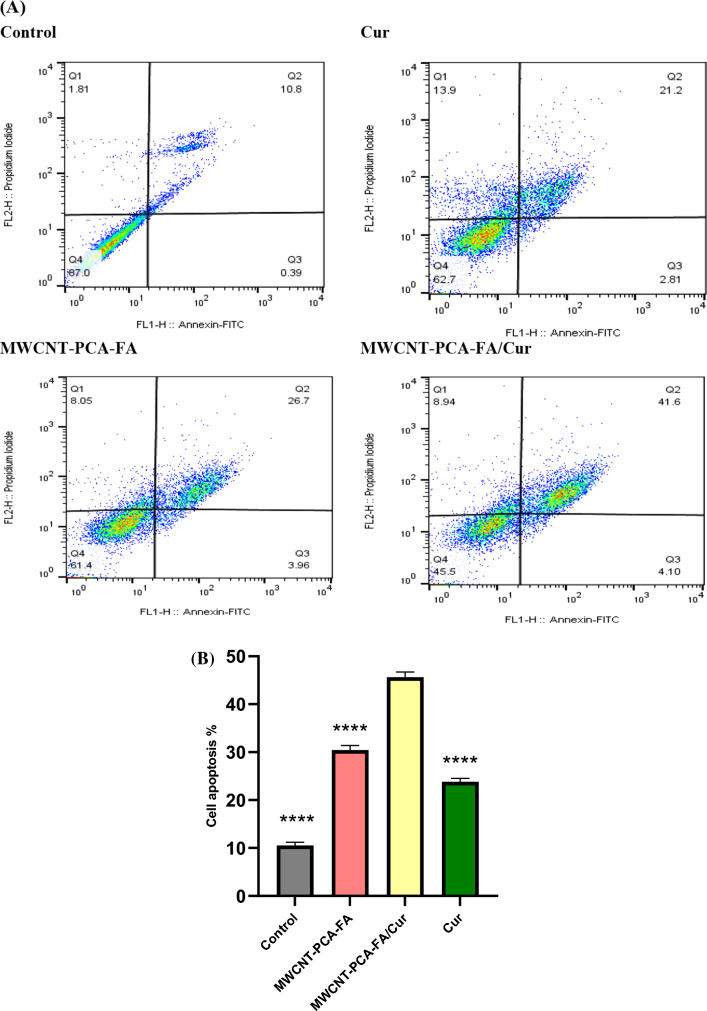
The apoptotic effect of MWCNT–PCA–FA, Cur, and MWCNT–PCA–FA/Cur treatment on B16F10 cells. (**A**) Annexin V + PI staining was used to calculate apoptosis. The percentages of early/late apoptosis and necrosis are represented by the numbers with dot plots. (**B**) The quantitative data are presented as the mean and standard deviation (S.D.) from three separate experiments. MWCNT–PCA–FA/Cur: ****p < 0.0001.

The initiation and progression of numerous human malignancies have been closely associated with dysregulated apoptosis^63^. One hallmark of cancer lies in the downregulation of apoptosis^64^. Notably, cell cycle arrest possesses the potential to induce cell apoptosis, a phenomenon that has been shown to play a pivotal role in both tumor development and therapeutic response. The evasion of apoptosis constitutes another characteristic shared by cancer cells, akin to aberrant cell cycle patterns. In this context, B16F10 cells were subjected to a 24-h exposure to free Cur, MWCNT–PCA–FA, and MWCNT–PCA–FA/Cur to ascertain whether the induced cell death by the synthesized compounds was intrinsically linked to apoptosis. To assess cell apoptosis post-exposure to these synthesized compounds, Annexin V and PI staining were judiciously employed. This staining method enabled the discrimination of viable cells (AVneg/PIneg), early-phase apoptotic cells (AVpos/PIneg), late-phase apoptotic cells (AVpos/PIpos), and necrotic cells (AVneg/PIpos). Notably, during apoptosis, phosphatidylserine translocates to the outer membrane, a phenomenon detectable via fluorochrome-labeled AV. Meanwhile, PI dye was employed for DNA staining, facilitating the distinction between live and deceased cells. As graphically represented in Fig. 7, after a 24-h treatment period, in comparison to the control group, MWCNT–PCA–FA robustly facilitated apoptosis within the B16F10 cell population. In contrast, free Cur engendered a relatively modest increase in apoptotic cells. The most striking combination of proliferative inhibition and cell death within B16F10 cells, however, was unequivocally achieved by MWCNT–PCA–FA/Cur. The apoptotic effect of free Cur, MWCNT–PCA–FA with and without Cur on B16F10 cells was meticulously evaluated after a 24-h exposure at a concentration of 1.25 µg/ml. These results were meticulously juxtaposed against the negative control. Notably, the most profound apoptotic effect was unmistakably exhibited by MWCNT–PCA–FA/Cur, closely trailed by the carrier, and followed by free Cur. This hierarchy in apoptotic efficacy is likely underpinned by the augmented bioavailability and solubility of Cur emanating from MWCNT–PCA–FA/Cur within the B16F10 cell line, surpassing that of the other compounds. MWCNT–PCA–FA/Cur nanoparticles unequivocally emerged as the most potent among all the compounds, affording a remarkable 45.7% of AV-positive cells, as opposed to MWCNT–PCA–FA's 30.66% and free Cur's 24.01% (Fig. 7A). Consequently, MWCNT–PCA–FA/Cur nanoparticles distinctly demonstrated the highest apoptotic percentage within the B16F10 cell population.

### Photothermal properties of MWCNT–PCA–FA/cur

**Figure 8:**
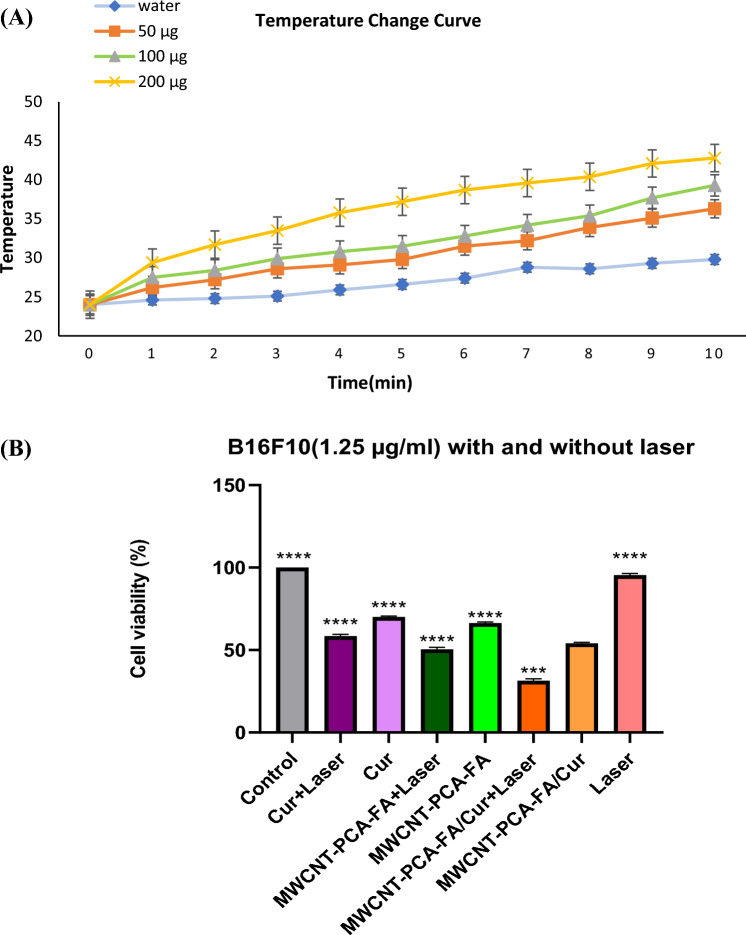
(**A**) The temperature changing curve of MWCNT–PCA–FA/Cur suspensions exposed to NIR laser for 10 min. (**B**) Cell viability study of Cur and the nanocomposites on B16F10 cell lines at optimum concentrations with and without laser radiation. The quantitative data are presented as the mean and standard deviation (S.D.) from three separate experiments. MWCNT–PCA–FA/Cur: ****p < 0.0001.

The obtained MWCNT–PCA–FA/Cur is anticipated to exhibit exceptional photothermal properties due to the presence of MWCNT. Thus, an assessment of the photothermal effect of MWCNT–PCA–FA/Cur was conducted by monitoring the temperature fluctuations of particle suspensions under NIR laser illumination. As illustrated in Fig. 8A, it is discernible that the temperature of MWCNT–PCA–FA/Cur suspensions (200 μg/ml) experienced a rapid surge, reaching 42.8 °C following irradiation with an 808 nm laser (1 W/cm^2^) for 10 min. In stark contrast, the temperature of deionized water subjected to identical treatment merely escalated to 29.8 °C after the same duration, conclusively indicating that NIR laser irradiation in isolation fails to induce a substantial photothermal effect.

### In vitro photothermal therapy of MWCNT–PCA–FA/Cur

This methodology empowers us to ascertain the prowess of the designed compounds in augmenting photothermal ablation of a wide spectrum of cancer cells. To this end, the B16F10 cell line, treated with MWCNT–PCA–FA/Cur, was subjected to NIR irradiation at 808 nm. The percentage of cell viability was determined through meticulous MTT assay. Initially, the B16F10 cell line underwent exposure to an 808 nm laser in isolation to evaluate cell viability, revealing no conspicuous cell mortality. Furthermore, the assessment of free Cur's toxicity in the presence and absence of NIR laser was conducted, yielding analogous mortality rates in both groups (below 50%). To gauge the impact of nanocomposites + laser, B16F10 cells were exposed to the optimal concentration of MWCNT–PCA–FA/Cur for 24 h, subsequently being irradiated at 808 nm. The optimal irradiation parameters, namely 10 min and a power density of 1.0 W/cm^2^, were determined to achieve nearly optimal cell ablation.

Remarkably, MWCNT–PCA–FA/Cur showcased the most potent cell mortality efficacy among all the compounds. As depicted in Fig. 8B, following MWCNT–PCA–FA/Cur + laser treatment, cell mortality reached an impressive 69%, notably surpassing the 49% and 42% observed for MWCNT–PCA–FA and free Cur + laser treatments, respectively.

## Conclusion

In this study, we meticulously engineered a multifaceted anti-cancer drug delivery system by amalgamating MWCNT–PCA–FA with free Cur. This resulting nanoplatform possesses a remarkable amalgamation of attributes: commendable biocompatibility, superior aqueous solubility, proficient photothermal conversion efficiency, steadfast protection of Cur from degradation, and the prompt release of Cur in an amorphous state within the formulation.

The crux of our innovation lies in the strategic incorporation of folate (FA) as a targeting ligand, which has unequivocally amplified the cellular uptake efficiency of this therapeutic nanoplatform. This enhancement, in turn, has translated into a substantial improvement in the use invitro non-invasive cancer ablation capabilities. Our investigative journey delved deeply into the intricacies of cellular processes, employing a repertoire of experimental techniques including cell cycle analysis, apoptosis assessment, and colony studies. Within the microenvironment of B16F10 cell cultures, MWCNT–PCA–FA/Cur emerged as a potent catalyst, bolstering the anti-tumor efficacy of Cur itself. This effect was further magnified through the integration of photothermal therapy mediated by MWCNTs, resulting in a synergistic impediment to tumor cell proliferation. The true novelty of our research lies in the confluence of these multifaceted advancements. We believe that our multifunctional therapeutic nanoplatform, MWCNT–PCA–FA/Cur, heralds a new era in cancer treatment, unlocking the potential for combination therapies that deftly leverage established therapeutic methodologies. This innovative approach has the potential to significantly elevate the efficacy of conventional anti-cancer treatments, paving the way for enhanced patient outcomes.

## Data Availability

The data that support the findings of this study are available from the corresponding author, upon reasonable request.
